# The Influence of pH Value on the Microstructure and Properties of Strontium Phosphate Chemical Conversion Coatings on Titanium

**DOI:** 10.3390/molecules28186651

**Published:** 2023-09-16

**Authors:** Guochao Gu, Yibo Li, Kangqing Zuo, Guiyong Xiao

**Affiliations:** 1 Key Laboratory for Liquid-Solid Structural Evolution and Processing of Materials, Ministry of Education, Shandong University, Jinan 250061, China; guochaogu@sdu.edu.cn (G.G.); liyibo_102@163.com (Y.L.); zuokangqing@foxmail.com (K.Z.); 2 School of Materials Science and Engineering, Shandong University, Jinan 250061, China

**Keywords:** titanium, phosphate chemical conversion, coating, strontium phosphate

## Abstract

Strontium (Sr) is a trace element in the human body that can promote bone formation and inhibit bone absorption. A conversion coating of strontium phosphate (Sr-P) on the surface of titanium (Ti) can improve its biological properties and has many potential applications in the fields of dentistry and orthopedics. In the present study, Sr-P coatings with SrHPO_4_ and Sr_3_(PO_4_)_2_ crystals on Ti are prepared by a phosphate chemical conversion (PCC) treatment and the effect of pH values on the properties of the Sr-P coatings is researched. The results prove that the phase composition, morphology, and corrosion resistance of the coated Ti vary according to the pH values of the PCC solution. The morphology of the conversion deposition on Ti changes from plat-like to cluster-like and then to homogeneous microcrystals as the pH value changes from 2.50 to 3.25. Only discrete SrHPO_4_ crystals are generated on the substrate at lower pH values, while relatively stable Sr_3_(PO_4_)_2_ and SrHPO_4_ crystals grow and subsequently form an integrated coating on the Ti as the pH exceeds 2.50. The cross-sectional morphologies and bonding strength of different coatings are also researched. The corrosion resistance of coated Ti improves compared with that of bare Ti because of the Sr-P coatings with a Sr_3_(PO_4_)_2_ phase. In addition, it is indicated that the Sr-P coatings on Ti can improve the adhesion and differentiation of BMSCs.

## 1. Introduction

Titanium (Ti) and its alloys have been of great interest in the fields of dentistry and orthopedics surgery owing to their suitable mechanical properties and good chemical resistance in vivo, with the help of their oxide films [1]. However, the corrosion resistance of Ti implants is greatly reduced by long-term interaction with body fluids. When it comes to surgical applications, the sustainability of Ti implants is in question because of toxic ions released by corrosion involving chloride ion and proteins in the harsh conditions in vivo. Furthermore, it is hard for Ti to achieve chemical bonds with bone due to its essential bioinertness [2]. Therefore, various surface modifications are applied to optimize the properties of Ti and its alloys [3]. Chemical conversion treatment is regarded as the simplest and most effective way to improve the surface properties of metals [4]. Because of its comparatively low cost and environmentally friendly characteristics, phosphate chemical conversion (PCC) treatment has been widely adopted to augment the corrosion resistance and bioactivity of metal implants [5]. In the last decade, PCC technology has been broadly used in surface modification for biomedical metallic materials such as magnesium, titanium, and zinc alloys [6,7]. In addition, some biofunctional cations, such as calcium (Ca^2+^), magnesium (Mg^2+^), zinc (Zn^2+^), and strontium (Sr^4+^/Sr^2+^) have also been commonly used as PCC-coated materials [8,9]. 

Strontium (Sr^2+^) is a bone-seeking trace element which is incorporated in bone in a similar way to Ca^2+^. It accounts for about 0.035% of the mineral components in the skeleton system [10]. It has been reported that the proper amount of Sr^2+^ can efficiently stimulate bone formation and enhance the mechanical properties of bone tissues because it can replace a moderate amount of Ca^2+^ in the lattice, which makes the array of atoms more compact and reduces lattice defects [11]. Moreover, the literature indicates that Sr^2+^ can improve corrosion resistance and stimulate bone formation by accelerating the differentiation of preosteoblasts and increasing the number of osteoblasts [12]. In addition, Sr can restrain the activity of osteoclasts and then decrease the number of osteoclasts to inhibit bone resorption [13]. Based on the evidence supplied above, Sr has promising prospects for applications in clinical therapy. The use of strontium phosphates (Sr-P) such as SrHPO_4_ and Sr_3_(PO_4_)_2_ as main compounds has attracted attention recently. In particular, SrHPO_4_ can be considered an ion exchanger biomaterial for holding both HPO_4_^2−^ and Sr^2+^ ions [14]. Sr_3_(PO_4_)_2_ also has been researched because it is a precursor of strontium apatite, a useful biomaterial [15]. 

Previous research results indicate that the parameters that affect the microstructure and properties of chemical conversion coatings include reaction temperature and time, as well as the pH value of the solution [16]. Of these, pH value is one of the most important factors in the formation of coating on metallic substrates, which can affect the formation rate and the properties of coatings such as coating mass, phase, and morphology. Studies in the literature have shown that metal phosphate ions can be deposited easily on a surface if the pH value of the reaction mixture exceeds its solubility limit [17]. Hence, it is important to investigate the effect of pH values of a PCC solution on the microstructure and properties of coatings on a Ti substrate.

However, few reports have focused on the fabrication of Sr-P coatings on Ti substrates, as well as the relationship between the coating properties and pH value of a reaction solution when using PCC treatment. Therefore, the aim of this study is to explore the feasibility of phosphate chemical conversion coating dopants with strontium and to investigate the effect of pH values on the microstructure and properties, such as anti-corrosion, bonding strength, and cytocompatibility, of these coatings on Ti.

## 2. Results

### 2.1. Phase Composition

**Figure 1:**
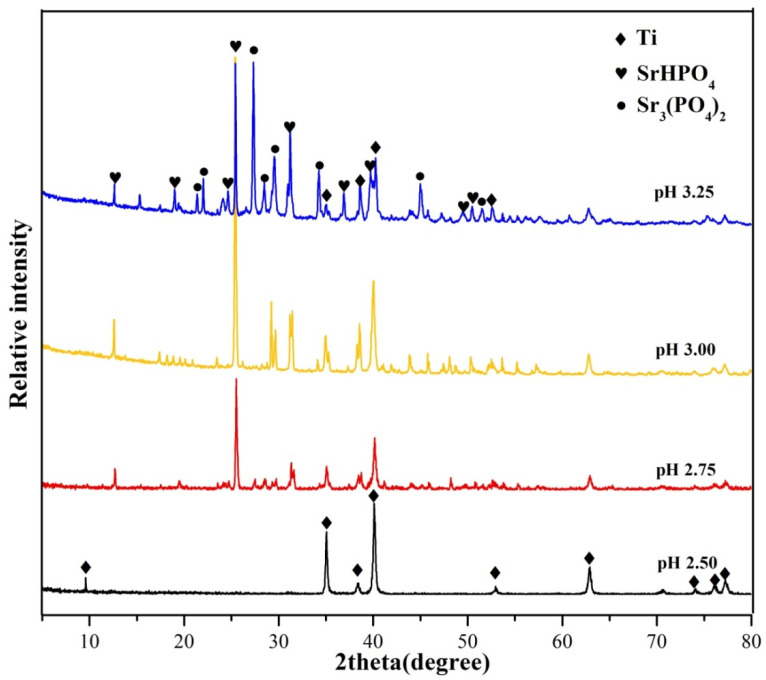
XRD patterns of conversion coatings on Ti with pH value from 2.50 to 3.25.

The XRD patterns of PCC coatings obtained with different pH values at 60 °C for 30 min are shown in Figure 1. The result of XRD patterns shows that effective conversion coating is not formed on the Ti surface when the pH is 2.50. However, SrHPO_4_ and Sr_3_(PO_4_)_2_ phases are detected on the Ti surface when the pH exceeds 2.50. Specifically, when the pH value is 2.75, SrHPO_4_ crystals begin to form on the substrate. In addition, some weak peaks of Sr_3_(PO_4_)_2_ appear on the coating when the pH = 2.75. Meanwhile, the peaks of Ti become weaker, indicating that substrate is covered by the conversion coating. As the pH value increases to 3.00, the relative peak intensity of the SrHPO_4_ becomes stronger, which indicates an increase in the covered area and coating thickness. The peaks of Sr_3_(PO_4_)_2_ appear clearly when the pH is 3.00. Figure 1 shows that as the pH increases to 3.25, strong diffraction peaks of Sr_3_(PO_4_)_2_ are detected. The peaks at 25.50°, 27.08°, and 31.36° are strong and sharp, implying good crystallinity of SrHPO_4_ and Sr_3_(PO_4_)_2_ [18].

### 2.2. Microstructure

**Figure 2:**
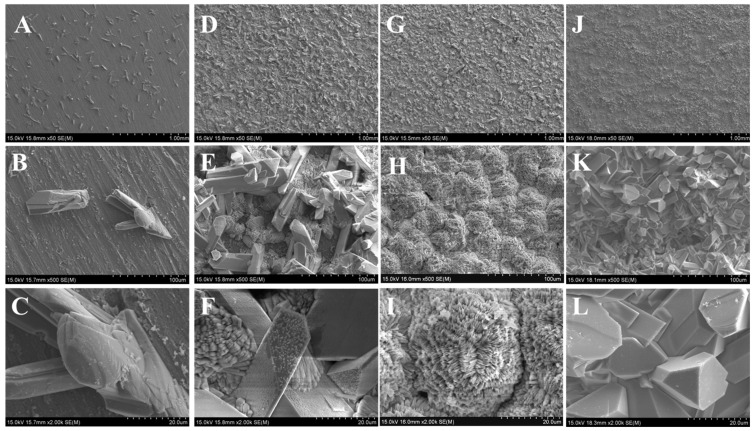
Surface morphology and corresponding high magnification images of conversion coatings by PCC treatment at various pH values. (**A**–**C**) 2.50, (**D**–**F**) 2.75, (**G**–**I**) 3.00, (**J**–**L**) 3.25. (**B**,**C**,**E**,**F**,**H**,**I**,**K**,**L**) are the high magnification images.

Figure 2 shows the surface morphology of Ti conversion coatings fabricated through PCC treatments at various pH values. The results show that only a few sporadic plate-like conversion crystals are discretely distributed on the Ti surface when the pH is 2.50. However, the continuous conversion coatings are formed as the pH exceeds 2.50. As the pH increases to 2.75, almost all the Ti substrate is covered by plate-like and cluster-like crystals. The morphology of the conversion crystals on Ti becomes finer and denser when the pH value increases to 3.25. Moreover, the cluster-like crystals are distributed between the plate-like crystals, as shown in Figure 2D–F. High magnification images (Figure 2E,F indicate the presence of microcrystals in the longitudinal axis direction of plate-like crystal, confirming the continuous growth along that way in the subsequent steps. In addition, the cluster-like crystals are incomplete, like numerous flakes being put together. As the pH value increases to 3.00 (Figure 2G), almost all the plate-like crystals disappear and are replaced by cluster-like crystals. Unlike the cluster-like crystals observed at pH 2.75, those at pH 3.00 are completed and compact. The images at high magnification (Figure 2H,I show that the cluster-like coating consists of compact flaky crystals with a nucleation core. As shown in Figure 2J, the Ti substrate is almost covered by bulk-like and tiny plate-like crystals with directionless growth when the pH is 3.25. These small crystals are evenly distributed on the Ti surface rather than forming clusters, as shown in Figure 2K,L.

Table 1 lists the compositions of the conversion crystals detected by EDS analysis. The PCC coatings at pH values ranging from 2.50 to 3.25 are mainly composed of C, P, Ti, Sr, and O, as shown in Table 1. At pH values of 2.50 and 2.75, it is seen that the quantity of Sr and P is nearly equal, which suggests that the crystals are in the SrHPO_4_ phase. The coating crystals exhibit Sr/P ratios of 1.12 and 1.25 at pH values of 3.00 and 3.25, respectively. In contrast, SrHPO_4_ and Sr_3_(PO_4_)_2_ exhibit ratios of 1.00 and 1.50, respectively, suggesting that the coating crystals formed at pH values of 3.00 and 3.25 are a mixture of SrHPO_4_ and Sr_3_(PO_4_)_2_ phases.

### 2.3. Bonding Strength

**Figure 3:**
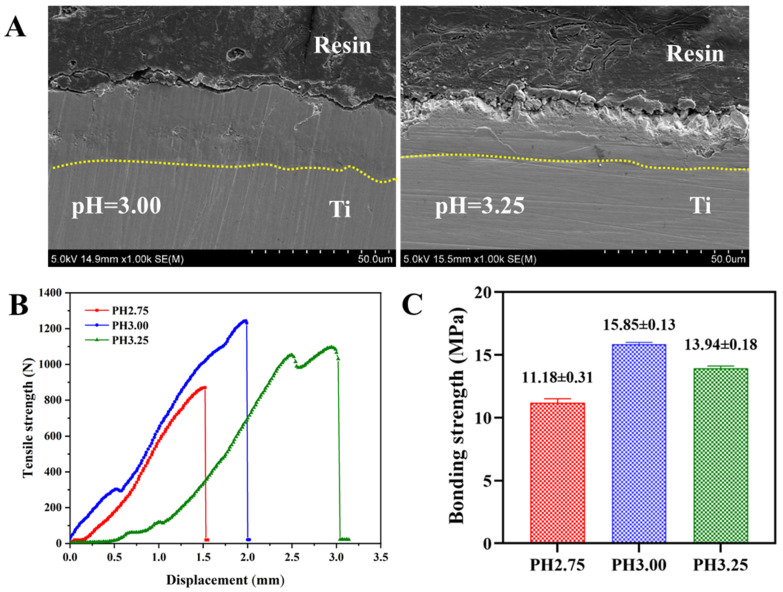
(**A**) The cross-sectional morphology of the Sr-P coatings on Ti, (**B**) tensile strength-displacement curves, (**C**) bonding strength of coatings with different pH values. The dotted yellow lines indicate the boundary between the coating and the substrate.

Figure 3 shows the cross-sectional morphology and the bonding strength of the Sr-P coatings on Ti at various pH values. The cross-section image of Ti at pH 2.5 is not given as no continuous coating was formed. Figure 3A illustrates that the interface of the Sr-P coatings (pH = 3.00 and 3.25) is well combined, with no obvious cracks between the coating and substrate. The thickness of the pH 3.00 coating is larger (~30 μm) than that of the pH 3.25 coating (~25 μm). The interior of the coating is relatively dense, except for some bulk-like crystals that exist on the surface of the Sr-P coating on Ti at pH 3.25, which is consistent with the results in Figure 2J. Figure 3B,C shows the tensile and bonding strength of the Sr-P coatings at various pH values. The tensile and displacement curves of the coating demonstrate that the tensile force of the Sr-P coating at pH = 3.00 was subjected to the greatest of 1244 N, while that of the coating at pH = 2.75 is only 872 N. However, the coating at pH = 3.25 exhibits the smallest slope of tensile force and displacement before failure. These results may be closely related to the thickness and microstructure of the Sr-P coatings. Among the different coatings, the pH = 3.00 coating has the highest bonding strength value of 15.85 ± 0.13 MPa. The bonding strength of the coating at pH = 2.75 is 11.18 ± 0.31 MPa. This can be attributed to the discontinuous structure of the coating, and its bonding strength primarily reflects the data of the acrylic adhesive. The bonding strength of the coating at pH = 3.25 is slightly reduced to 13.94 ± 0.18 MPa due to the presence of bulk-like crystals on its surface.

### 2.4. Corrosion Characteristics

**Figure 4:**
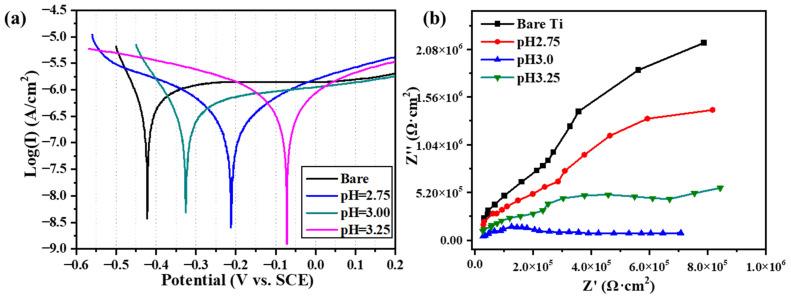
The electrochemical properties of bare and coated Ti samples fabricated with different pH values. (**a**) Potentiodynamic polarization, (**b**) Nyquist plots.

Figure 4 presents the potentiodynamic polarization curves of bare Ti and PCC coated samples treated by different pH values in SBF. The parameters of the electrochemical corrosion of different samples are listed in Table 2. Due to only a few crystals being observed on the surface of Ti at pH = 2.50 and no coating being formed at all, its electrochemical data are not presented in this part. The results clearly illustrate that the open circuit potential (*E_corr_*) value is a function of processing pH values. And the corrosion current density (*I_corr_*) of the samples is improved with an increase in the pH value of the PCC solution. The *E_corr_* of the coated Ti samples is oppositely decreasing, but the coating with pH = 3.00 is an exception. This is related to its flaky-like microstructure and phase composition. In addition, the bare Ti shows the strongest *E_corr_* (−0.426 V) and higher *I_corr_* (42.67 × 10^−8^ A/cm^2^), compared to the other coated Ti substrates. Table 2 shows that the R_p_ values of coated Ti are greater than that of the bare sample, indicating that the SrHPO_4_ phosphate coatings can improve the anti-corrosion properties in comparison to the bare Ti sample. Meanwhile, the coating with pH = 3.00 exhibits the highest *R_p_* value and *E_corr_* among the other samples, which indicates that it has the best corrosion resistance. These results are closely related to the microstructure of the conversion coatings on the Ti surface.

### 2.5. Cytocompatibility

**Figure 5:**
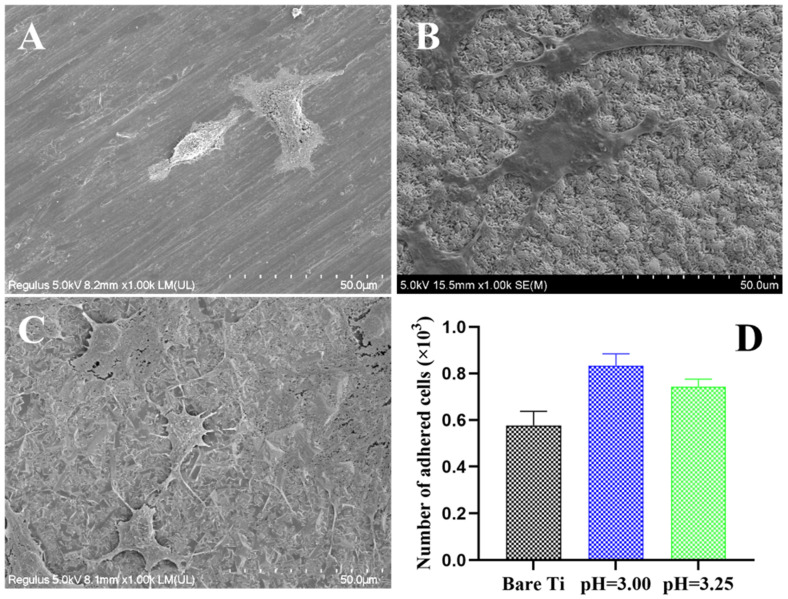
Morphologies and adhering number of BMSCs on bare and coated Ti samples with different pH values after culture for 48 h. (**A**) Bare Ti, (**B**) pH = 3.00, (**C**) pH = 3.25, and (**D**) the number of adhered cells.

Figure 5 shows the morphologies and cell number of BMSCs adhering to the surface of Ti substrates after being cultured for 3 days. Due to the incomplete structure of the coatings on Ti obtained at pH 2.50 and 2.75, only two samples with pH 3.00 and 3.25 are chosen for biological tests in this section. The results illustrate that BMSCs spread well and present elongated pseudopodia on the two kinds of coated Ti implants, while the cells on the bare Ti sample appear approximately spherical in shape (Figure 5A–C). Additionally, the CCK-8 result proves that the number of adhered cells on the coated Ti surfaces is higher compared to the bare Ti. The cells on the Sr-P coatings with pH = 3.00 have the most benefits for the differentiation of BMSCs, as shown in Figure 5D. These data demonstrate that both the phase composition and microstructure of the conversion coating on Ti have an impact on the adhesion and differentiation of cells. In addition to the fine crystal structure of the coating, its mixture phases of the SrHPO_4_ and Sr_3_(PO_4_)_2_ phases (as shown in Figure 1) can also promote cell differentiation.

## 3. Discussion

This present study aims to provide a simple, effective, and anti-corrosion Sr-P chemical conversion coating by the PCC method to improve the properties of Ti substrates. Electrochemical reactions will proceed during the PCC treatment, which includes obtaining electrons around the Ti surface and losing electrons at the cathode [19]. In PCC processing, all the phosphate in the solution is mainly treated as H_2_PO_4_^−^, as the pH changes from 2.50 to 3.25. When the discharge of hydrogen ions occurs at the cathode, the regional pH value near the Ti substrate increases, which will result in the formation of HPO_4_^2−^ and PO_4_^3−^, as described in Equation (1) [20]. As the pH values around the Ti substrate continue to rise, SrHPO_4_ will first precipitate from the PCC solution due to its lower solubility. The reaction in Equation (2) will happen and Sr_3_(PO_4_)_2_ will form with an increase in the pH value.

H_2_PO_4_^−^ ↔ HPO_4_^2−^ + H^+^ ↔ PO_4_^3−^ + 2H^+^ (1)

Sr^2+^+ HPO_4_^2−^→SrHPO_4_↓ (2)

3Sr^2+^+2PO_4_^3−^→Sr_3_(PO_4_)_2_↓ (3)

As shown in Figure 2, the number of crystals increases and their size reduces with the augment of the pH value, whether the morphology of crystals is mainly plate-like at pH 2.50 and 2.75, chiefly fine flaky-like at pH 3.00, or small bulk-like at pH 3.25. This result illustrates that the morphology of depositions on the Ti substrate is markedly concerned with the H+ ions in the chemical solution, which is consistent with the results of some of the literature [21,22]. Gashti et al. have shown that the morphology of SrHPO_4_ obtained via 0.80 M Na_2_HPO_4_ and 1 M SrCl_2_ is denser and more compact than that obtained via 0.50 M Na_2_HPO_4_ and 0.50 M SrCl_2_ [23]. A reasonable explanation for these phenomena is that relatively stable and low-saturated degrees always achieve large-sized single crystals. This is because low saturated degrees cannot generate crystal nuclei spontaneously but can only make crystals grow along the original nucleus (or crystal) until the completed crystal is formed [24]. In other words, when the solution pH increases, it is easier for H_2_PO_4_^−^ to transform to HPO_4_^2−^, the ingredient of SrHPO_4_, which leads to a higher saturated degree of SrHPO_4_. Naturally, an increase in crystal sites is accompanied by a decrease in size. Therefore, the crystals of coating at pH 2.50 are large and plate-like, whereas those at pH 3.00 are cluster-like and denser. Specifically, the reason why both cluster-like and large plate-like crystals are formed is that the formations of large plate-like crystals augment the pH value of the solution, which further accelerates the formation of H_2_PO_4_^−^ and the saturated degree. In addition, those fine flaky crystals that originally clustered at pH 3.00 distribute evenly on the Ti surface. Since SrHPO_4_ is a triclinic crystal and a ≠ b ≠ c, α ≠ β ≠ γ, SrHPO_4_ crystals can grow along any direction, and the irregular phases observed in the figure are mainly composed of SrHPO_4_. Meanwhile, Sr_3_(PO_4_)_2_ matches the characteristics of hexagonal crystal, resulting in a more regular shape and structure in Figure 2J [25].

The interface characteristics and bonding strength of coatings on Ti substrate are the key factors for assessing coating quality. As shown in Figure 3, the interface of Sr-P coatings is well combined with the substrate and no obvious cracks appear, suggesting good bonding strength. The thickness of the coating is related to the crystal structure of Sr-P. Coating with cluster-like SrHPO_4_ distribution at pH 3.00 tends to form a thicker aggregate. According to the literature, the fracture of the coating on metal substrate can occur in four stages as follow as: the cohesive failure in the interior of the adhesive, the adhesive failure in the adhesive-coating interface, the coating failure in the interior of the coating, and the bonding failure in the coating-substrate interface, respectively [26,27]. As shown in Figure 3C, the bonding strength of the pH 2.75 coating is relatively low (11.18 ± 0.31 MPa), which maybe because the structure of this coating is discontinuous; the fracture type is the cohesive failure in the interior of the adhesive. So, its bonding strength primarily reflects the data of the acrylic adhesive. For the complete coatings on Ti, the bonding strength is mainly related to the initial characteristics of the substrate, and the thickness, microstructure, phase composition, and other properties of the coatings [27,28,29]. In this work, the pH 3.00 coating has a cluster of flaky-like SrHPO_4_ crystals with micro/nanostructure, which can improve the coating failure in the interior of the coating. On the other hand, the pH 3.25 coating is composed of SrHPO_4_ and Sr_4_(PO_4_)_2_ crystals with bulk-like and tiny plate-like crystals. These bulk-like crystals may render the coating more brittle and reduce the bond strength, despite its smaller thickness.

*I_corr_* and *E_corr_* derived from the measurements of the specimens are used to evaluate the protective property of the coatings. Bare Ti shows good anti-corrosion property because of the chemically stable passive film on the surface of Ti, as shown in Figure 4. The strongest *E_corr_* and higher *I_corr_* values in the electrochemical test mean the coatings have better anti-corrosion property [30]. The coated Ti substrates have better corrosion resistance compared with the bare Ti, as the Sr-P conversion coatings are formed during PCC processing. The sample with the pH = 2.50 has only the sporadic plate-like SrHPO_4_ crystals, as a precursor of the Sr_3_(PO_4_)_2_ phase [31] is generated, as shown in Figure 1. Since no continuous coating is formed at pH = 2.50, there is a dual effect of crystal dissolution and oxide film protection in SBF. When this sample is incubated in SBF solution, the passive film TiO_2_ still plays a major role in anti-corrosion. However, the SrHPO_4_ and Sr_3_(PO_4_)_2_ crystals on Ti can influence the electrochemical data in Figure 4 when the pH values exceed 2.50. The *E_corr_* and *I_corr_* data lack regularity due to the non-integrity of the coating and the variation in the microstructure of Sr-P crystals. Therefore, it is necessary to further study the *R_p_* values to evaluate the corrosion resistance of the coating [32]. As the pH value increases, the relatively stable Sr_3_(PO_4_)_2_ crystals grow and subsequently form a coating on Ti substrate, thus improving the corrosion resistance of the samples. However, the coating of pH = 3.00 has the compact cluster of flaky-like crystals, which can affect its electrochemical data. Further rules and reasons will be researched in future investigation.

The biological response of the cells around the implant is the result of the combined effect of the phase composition and the microstructure of conversion coatings. So, their optimization should be considered comprehensively when designing surface modification on Ti. As shown in Figure 5, the conversion coatings with pH 3.00 and 3.25 have obvious micro/nano microstructure, which can provide excellent physical conditions for the adhesion and differentiation of BMSCs. Hulshof’s research proved that the fate of cells can be determined through designing the surface microstructure and specific physicochemical properties [33]. The flaky- and bulk-like crystals on the Sr-P coatings allow the cell pseudopods to extend and embed into the gaps between the crystals, thus promoting cell adhesion and differentiation. Compared to the pH = 3.25 coating, the pH = 3.00 coating has cluster flaky crystals with nanostructure, which can better promote cell bioactivity, as shown in Figure 5D. Apart from the microstructure, the phase composition of the coating also significantly affects the biological behavior of the BMSCs [12,34]. Strontium phosphates has better bioaffinity and improve the proliferation of bone stem cells. Additionally, studies have reported that higher doses of Sr (above 9 mM) induce apoptosis in rabbit’s mature osteoclasts by stimulating the calcium-sensing receptors [35]. Under the influence of the culture medium, both the Sr-P conversion coatings can release the element Sr, which can significantly improve the differentiation ability of BMSCs, as shown in Figure 5D. In addition, Sr may indirectly inhibit osteoclast formation and bone resorption by regulating the expression of OPG [36]. For the coatings with a pH = 3.00, there are more SrHPO_4_ and Sr^2+^ ions released, resulting in improved cell differentiation.

## 4. Materials and Experimental Methods

### 4.1. Surface Pretreatment

Commercial Ti was processed into Ø10 mm × 3 mm cylinders as substrates. All the Ti samples were polished to obtain homogeneous roughness. Then, the substrates were degreased in 80 g/L sodium hydroxide (NaOH) solution at 50 °C for 15 min. Next, the cylinders were etched with 2.00% hydrofluoric acid (HF) at room temperature for 15 s. Finally, the samples were immersed in 3.00 g/L colloidal titanium phosphate to increase the nucleation points on the Ti surface. The bare Ti disks were used as control.

### 4.2. Phosphate Chemical Conversion

The PCC treatment was similar to that reported previously [6]. Briefly, pretreated Ti specimens were put into the PCC solution which contained 0.20 mol/L NaH_2_PO_4_, 0.40 mol/L Sr (NO_3_)_2_, 2.00 g/L NaNO_2_, and 5.00 g/L Iron powder. After aging for 24 h, the pH value of the PCC solution was adjusted to 2.50, 2.75, 3.00, and 3.25, respectively, using sodium hydroxide (NaOH, 5.00 mol/L) or phosphoric acid (H_3_PO_4_, 7.00% *v*/*v*). Finally, the pretreated Ti samples were incubated in the PCC solution for 30 min at 60 °C to research the effect of different pH values on the microstructure and properties of coated samples.

### 4.3. Bonding Strength

According to the ASTM C633-01 standard [37], the bonding strength of samples was tested using a mini-type universal testing device (WDW-5, STAR, Jinan, China) with a maximum capacity of 5 kN. Before testing, the coated-Ti samples on both sides were bonded with two stainless-steel cylinders using acrylic adhesive, and then cured at room temperature for 24 h. Then, a tensile load was applied to the samples at a rate of 1.00 mm⋅min^−1^ until fracture occurred. The bonding strength of samples was calculated as the ratio of the maximum stripping load to the surface area of coated specimens before failure. The average values of three stable datasets were selected for each group of samples.

### 4.4. Electrochemical Measurements

Electrochemical impedance spectroscopy (EIS) of the PCC coatings was carried out on an automatic laboratory corrosion measurement system (PARSTAT 2273, Shanghai, China). A classical three-electrode cell was set up in the simulated body fluid (SBF) at a scan rate of 2 mV/s^−1^. The saturated calomel electrode (SCE), platinum, and the sample coupon with 1 cm^2^ exposed area were used as reference, counter, and working electrodes in the three-electrode cell (CHI660E, Shanghai, China), respectively. The Tafel polarization curve was calculated using a constant voltage scan rate. And then, the equilibrium potential (*E_corr_*), the corrosion current (*I_corr_*), and the anode/cathode Tafel slope (*β_a_/β_c_*) were deduced. The polarization resistance (*R_p_*) was calculated according to the Stern–Geary equation [38].

$$R_{p}=\frac{\beta_{a}\cdot \left|\beta_{c}\right|}{2.303\cdot I_{corr}\cdot \left(\beta_{a}+\left|\beta_{c}\right|\right)}$$

### 4.5. Cell Culture

Bone marrow-derived stem cells (BMSCs) were cultured in fresh Dulbecco’s modified Eagle’s medium (DMEM, Gibco, Grand Island, NY, USA) containing 10% (*v*/*v*) fetal bovine serum (FBS, Gibco, Grand Island, NY, USA) and 1% penicillin/streptomycin in a humidified atmosphere at 37 °C and 5% CO_2_. The polished Ti and its coatings were sterilized using ultraviolet for 1 h. Then, the BMSCs were seeded onto the samples in 24-well plates at a density of 2 × 10^4^ cells/well. The proliferation rates of the BMSCs cells grown on different samples were assessed using a Cell Counting Kit-8 (CCK-8 kit, Dojindo Molecular Technologies, Tokyo, Japan). The BMSCs cells with three replicates were seeded into a 24-well plate and pre-incubated for 48 h to allow for complete adherence before conducting the CCK-8 assay. All the CCK-8 values were normalized to the control, which represents 100% cell viability.

### 4.6. Characterization of Samples

The morphologies of the PCC samples and BMSCs on the coated Ti were tested using a scanning electron microscope (FE-SEM, Hitachi SU-70, Tokyo, Japan) equipped with an energy dispersive spectrometer (EDS, Hitachi, Tokyo, Japan). All the samples were sputtered by nano golden particles before testing. The phase composition of the coatings was examined by an X-ray diffractometer (XRD, Rigaku D/max-γB, Rigaku, Tokyo, Japan) using a Cu-Kα radiation operated at 40 kV and 100 mA, with a scan rate of 4°/min and a scan step of 0.02° from 10° to 80°.

## 5. Conclusions

The Sr-P conversion coatings were successfully prepared on Ti substrates using PCC processing in this work. The phase composition, morphology, corrosion resistance, and cytocompatibility of coated Ti varied with different pH values. At pH 2.50, only a few sporadic plate-like SrHPO_4_ crystals were generated on the substrate. As the pH values increased, relatively stable SrHPO_4_ and Sr_3_(PO_4_)_2_ crystals grew and subsequently formed a continuous coating on Ti substrate. The morphologies of conversion deposition on Ti present a structure from plate-like to flaky-like, and then evenly bulk-like microcrystals, as pH value changes from 2.50 to 3.25. The continuous Sr-P coatings have good interface bonding with Ti substrate. The coating at pH = 3.00 exhibits the highest bonding strength of 15.85 ± 0.13 MPa. The corrosion resistance of coated Ti improved due to the increase in the Sr_3_(PO_4_)_2_ phase in the coatings. Additionally, the coatings with pH = 3.00 exhibit the best anti-corrosion property. Moreover, the Sr-P coatings also possessed good cytocompatibility and could promote the differentiation of BMSCs. The Sr-P coating with pH = 3.00 exhibited better cell differentiation due to its microstructure and phase composition.

## Figures and Tables

**Table 1:** EDS analysis of conversion crystals by PCC treatment at various pH values.

pH Value	O	P	Sr	Ti	C	Sr/P
2.50	67.59	9.56	10.10	2.81	9.94	1.06
2.75	62.13	13.52	13.85	----	10.50	1.02
3.00	70.17	14.02	15.68	0.13	----	1.12
3.25	57.52	13.20	16.52	----	12.76	1.25

Note: Data in this table means atom%. “----” means this element was not checked out or was not selected.

**Table 2:** Electrochemical corrosion parameters determined by potentiodynamic polarization curves of the bare Ti and coated samples with different pH values. Data are shown as mean ± SD, *n* = 3.

Sample	*E_corr_* (V)	*I_corr_* (×10^−8^ A/cm^2^)	*β_a_* (V·dec^−1^)	*−β_c_* (V·dec^−1^)	*R_p_* (×10^4^ Ω·cm^2^)
Bare Ti	−0.426 ± 0.006	42.67 ± 4.35	0.129 ± 0.007	0.107 ± 0.005	11.230 ± 0.675
pH = 2.75	−0.212 ± 0.009	28.32 ± 6.24	0.221 ± 0.010	0.179 ± 0.019	15.163 ± 0.022
pH = 3.00	−0.325 ± 0.011	30.04 ± 4.54	0.399 ± 0.006	0.088 ± 0.013	24.122 ± 0.286
pH = 3.25	−0.072 ± 0.016	53.89 ± 3.46	0.445 ± 0.001	0.215 ± 0.002	16.026 ± 0.954

## Data Availability

The data presented in this study are available on request from the corresponding author. The data are not publicly available due to issues related to the proprietary rights.
